# Covid 19 vaccines and the misinterpretation of perceived side effects clarity on the safety of vaccines

**DOI:** 10.37796/2211-8039.1371

**Published:** 2022-09-01

**Authors:** Raymond D. Palmer

**Affiliations:** a Full Spectrum Biologics, South Perth, WA, 6151, Australia; b Full Spectrum Biologics, WA, 6102, Australia

**Keywords:** Covid 19, Vaccines, Side effects, Misinterpretation, Ischemia, Stress, Cardiovascular

## Abstract

In the era of Covid 19 and mass vaccination programs, the anti-vaccination movement across the world is currently at an all-time high. Much of this anti-vaccination sentiment could be attributed to the alleged side effects that are perpetuated across social media from anti-vaccination groups.

Fear mongering and misinformation being peddled by people with no scientific training to terrorise people into staying unvaccinated is not just causing people to remain susceptible to viral outbreaks, but could also be causing more side effects seen in the vaccination process. This brief review will offer data that may demonstrate that misinformation perpetuated by the anti-vaccination movement may be causing more deaths and side effects from any vaccine.

A mini review of published literature has been conducted and found that mental stress clearly causes vasoconstriction and arterial constriction of the blood vessels. Therefore, if subjects are panicked, concerned, stressed or scared of the vaccination, their arteries will constrict and become smaller in and around the time of receiving the vaccine. This biological mechanism (the constriction of veins, arteries and vessels under mental stress) is the most likely cause for where there has been blood clots, strokes, heart attacks, dizziness, fainting, blurred vision, loss of smell and taste that may have been experienced shortly after vaccine administration. The extreme mental stress of the patient could most likely be attributed to the fear mongering and scare tactics used by various anti-vaccination groups.

This paper does not aim to rule in or out every side effect seen, but it is highly likely that many apparent side effects seen shortly after a subject has received a vaccine could be the result of restricted or congested blood flow from blood vessel or arterial constriction caused by emotional distress or placebo based on fear around vaccines.

## 1. Introduction

Vaccines introduced in late 2020 or early 2021 were closely watched, scrutinised, and monitored by the world’s mainstream population due to their fast to market delivery. Subsequent health concerns were quickly made public across social media and news outlets driving further vaccine hesitancy. One of the most common health concerns was that various types of Covid 19 vaccines were causing strokes or blood clots. The science for the vaccines causing blood clots has not been found, but other causes for this cascade from vaccines to blood clotting events may be found in existing medical literature.

Covid 19 vaccines use many of the same ingredients that have been safely used for many years, with the only major difference being the mRNA [1,2]. However, anti-vaccination sentiment and side effects are at an all time high, and this may point to a statistical significance.

Vaccines include antigens that produce an immune response which is adept at providing protection from disease [3]. However, reactogenicity from vaccines is very rare according to Herve et al. and mostly associated with mild irritations or other discomfort at the site of injection. Once vaccine antigens enter the body, they are distinguished as pathogens by the body’s immune system, the pathogen-associated molecular patterns (PAMPs) or damage-associated molecular patterns (DAMPs), pattern-recognition receptors (PRRs), including Toll-like receptors (TLR) that are located on peripheral circulating immune cells [3,4].

Even though the likelihood of mental stress causing strokes, heart attacks or blood clots may at first appear unlikely, a brief investigation of current medical literature clearly shows that simple tasks under clinical observing conditions such as public speaking can induce serious adverse outcomes [5]. Krantz et al. demonstrated that subjects with ischemia from mental stress experienced cardiac episodes more frequently than subjects without mental stress ischemia (8 of 34; 23%; p = 0.048).

Mental stress-induced myocardial ischemia (MSIMI) is a condition where blood flow to the heart is restricted due to emotional distress. MSIMI has been found to be more severe in females when peripheral blood vessels are constricted [6]. If MSIMI results in ischemia, it can also double the chance of a heart attack or death in subjects where heart disease is present [7]. Jiang et al. found a significant increase in nonfatal and fatal cardiac events in subjects with MSIMI.

It has been found that the level of microvascular constriction but not the angiographic burden of coronary artery disease (CAD) is correlated with MSIMI [8]. Patients with CAD and exercise induced ischemia (EII) with the existence of MSIMI were highly predicted to undergo a loss of life event [9].

Visceral arteries are also implicated in constriction from mental stress. Notably the renal artery showed decreased blood flow during mental stress testing [10]. The superior mesenteric artery (SMA) did not display any significant difference according to Hayashi et al. The findings of renal artery constriction also may lead into serious downstream kidney events. This data clearly indicates that mental stress can prevent blood flow far beyond the cardiovascular system inducing many other aberrations.

Adverse cardiovascular events that were reported from Covid 19 vaccines have been monitored closely by The World Health Organisation (WHO) [11]. Of those events, palpitations (717(14.74)), increased heart rate (439 (9,03), flushing (592(12.17) and tachycardia (798 (16.41)) were all reported as having the highest rate of incidence. However, Kaur et al. does not find any causality from the vaccines listed. Furthermore, restricted blood flow or blockages caused by MSIMI inducing vasoconstriction could be the smoking gun in all the aforementioned conditions such as palpitations, increased heart rate, flushing and tachycardia [12,13].

Vaccines monitored by Kaur et al. were Comirnaty (BNT162b2), Moderna COVID-19 Vaccine (mRNA1273), COVID-19 Vaccine AstraZeneca (AZD1222); also known as Covishield, Sputnik V, COVID-19 Vaccine Janssen (JNJ-78436735; Ad26.COV2.S), CoronaVac, BBIBP-CorV, Epi-VacCorona, Convidicea (Ad5-nCoV), Covaxin, CoviVac, ZF2001.

Moreover, vasoconstriction could also result in hyperpnea, postural faint, light headedness or dizziness which have all been included as possible side effects from the Covid 19 vaccines [14,15]. Post vaccine smell and taste disorders have also been implicated as side effects of Covid 19 vaccines, however both these disorders may be attributed to blood flow disorders from mental stress induced vasoconstriction [16].

The litany of suspected or perceived side effects discussed here from Covid 19 vaccines correlates firmly with well-established vasoconstriction disorders where blood flow is reduced or blocked completely. MSIMI is found in 70% of people with CAD [17], and it is predicted that approximately 16.3 million Americans above the age of twenty have CAD. Notably The American Heart Association (AHA) reports that approximately 82.6 million people in the United States have some form of cardiovascular disease [18]. When MSIMI is combined with these conditions, it presents a further aggravated risk for mortality.

The data presented herein, poses an interesting question, is the fear mongering around vaccines causing many of these perceived side effects by inducing unnecessary stress in vulnerable people? Is the movement and character of anti-vaccination information that may strike fear into the general population causing anxiety and vascular constriction resulting in pathologies such as dizziness, hypernea, fainting, blood clotting, stroke and heart attack? The science discussed here clearly establishes that anxiety and fear causes vasoconstriction disorders, and that a particular movement that is trying to save people with a profound lack of scientific and medical training (the anti-vaccination movement) from vaccine side effects may actually be the entity causing the majority of side effects.

Sullivan et al. had demonstrated that MSIMI was found to be more dangerous in females when peripheral blood vessels were constricted. When females underwent a tonometry exam the average ratio was associated at 0.11–0.35, just over a threefold ratio [6]. The Centre for Disease Control (CDC) also found that there was approximately between a three and fourfold increase in females reporting adverse side effects than men from the Covid 19 vaccine [19]. The numbers reported from the CDC were 4296 adverse side effects from females, and 1056 from men. The parallel in this data is quite clear, and may profoundly exonerate Covid 19 vaccines as ground zero for the perceived side effects and implicate the well established and studied condition of MSIMI and other blood flow conditions as the smoking gun.

Apart from MSIMI and other cardiac impairments discussed here, the placebo effect is also a strong marker in potential side effects, as the belief in detrimental side effects (the nocebo effect) can cause detrimental side effects [20]. It has also been shown that the placebo effect can be so powerful that it can affect end-organ functions that are controlled by the autonomic nervous system [21]. Both the placebo and nocebo effect are both noted here due to MSIMI being caused by mental stress, that is the connection between mental state and biological disorder which is already well established across the literature. This shows major cause for concern where fear mongering around vaccines is being perpetuated, as those with expectations of getting adverse side effects may increase their risk of experiencing adverse side effects [22].

Obesity may also play a role in poor outcomes for Covid 19 vaccines [23]. Obese subjects also appear to be at higher risk of MSIMI [24] which is consistent with this paper’s findings. An increase in adverse reactions was also found in obese subjects when using the Pfizer vaccine [25]. Obesity or poor arterial health may heighten the chances of a vaccine side effect.

## 2. Conclusion

This mini review finds that subjects with a history of heart disease, obesity, poor health combined with extreme stress or fear of vaccines should visit their medical practitioner and discuss the use of therapies or medications such as vaso or arterial dilators or possibly anticoagulants prior to their vaccines, as these measures under professional guidance may assist in maintaining healthy blood flow through a subject’s system and may offer benefits to ensure adverse reactions from underlying health conditions are not confused with adverse reactions from vaccines.

All data or claims of adverse reactions from vaccines should first be weighed against a subject’s health history with a focus on their vascular and arterial systems, cardiologic fitness and propensity for mental stress induced ischemia.

This brief review is not exhaustive but finds that it is highly probable that many adverse reports from recent vaccines are associated with vasoconstriction in conjunction with MSIMI or CAD.

This paper also presents the opportunity for governments to peer back into the claims of adverse vaccine side effects and weigh up the volume of existing health conditions that many of those subjects may have had. If it can be established in a high volume of cases of apparent side effects that CAD, HD, MSIMI or EII were present, then the adverse reactions can be laid against emotional distress or anxiety as opposed to the vaccines. The cause or source of that emotional distress and fear must then be investigated, recognised, and managed for future vaccination programs. Humanity on average has experienced a viral outbreak every two years for the last decade. So, managing this alarmism over perceived vaccine side effects is paramount in delivering fast to market solutions for future vaccination programs.

### 2.1. Limitations of study

This review is limited by primarily focusing on vasoconstriction conditions caused by a stress response, and also by a lack of large-scale clinical trial studies to determine whether using novel combinations of vasodilators or anticoagulants with vaccines could reduce vaccine side effects, which may also assist in clarifying whether side effects were emanating from vaccines or conditions such as MSIMI. Further investigation into whether side effects could be attributed as a stress response is required.
